# Sensitivity of Multiphase Pseudocontinuous Arterial Spin Labelling (MP pCASL) Magnetic Resonance Imaging for Measuring Brain and Tumour Blood Flow in Mice

**DOI:** 10.1155/2018/4580919

**Published:** 2018-11-07

**Authors:** Jessica Buck, James R. Larkin, Manon A. Simard, Alexandre A. Khrapitchev, Michael A. Chappell, Nicola R. Sibson

**Affiliations:** ^1^Cancer Research UK and Medical Research Council Oxford Institute for Radiation Oncology, Department of Oncology, University of Oxford, OX3 7LE, Oxford, UK; ^2^Institute of Biomedical Engineering, University of Oxford, Old Road Campus Research Building, Oxford OX3 7DQ, Oxford, UK

## Abstract

Brain and tumour blood flow can be measured noninvasively using arterial spin labelling (ASL) magnetic resonance imaging (MRI), but reliable quantification in mouse models remains difficult. Pseudocontinuous ASL (pCASL) is recommended as the clinical standard for ASL and can be improved using multiphase labelling (MP pCASL). The aim of this study was to optimise and validate MP pCASL MRI for cerebral blood flow (CBF) measurement in mice and to assess its sensitivity to tumour perfusion. Following optimization of the MP pCASL sequence, CBF data were compared with gold-standard autoradiography, showing close agreement. Subsequently, MP pCASL data were acquired at weekly intervals in models of primary and secondary brain tumours, and tumour microvessel density was determined histologically. MP pCASL measurements in a secondary brain tumour model revealed a significant reduction in blood flow at day 35 after induction, despite a higher density of blood vessels. Tumour core regions also showed reduced blood flow compared with the tumour rim. Similarly, significant reductions in CBF were found in a model of glioma 28 days after tumour induction, together with an increased density of blood vessels. These findings indicate that MP pCASL MRI provides accurate and robust measurements of cerebral blood flow in naïve mice and is sensitive to changes in tumour perfusion.

## 1. Introduction

Reliable and accurate quantification of cerebral blood flow (CBF) by noninvasive methods, such as magnetic resonance imaging (MRI), is of critical importance in many neuropathologies. The measurement of tumour blood flow, for example, is key to both treatment planning and predicting response. Tumour perfusion is widely used as an indicator of tumour microenvironment, stage, and progression, and greatly influences treatment. Clinical studies have shown that tumour perfusion and, consequently, oxygenation are highly correlated with clinical outcome [1–3]. Critically, the increased use of antiangiogenic drugs and vascular normalising therapies in conjunction with radiotherapy to treat tumours has resulted in a need for improved quantitative, noninvasive imaging measurements of vascular parameters to monitor treatment response.

The MRI method arterial spin labelling (ASL) is often used to image cerebral blood flow (CBF), as it requires no exogenous contrast agent and is easily implemented on clinical scanners. Moreover, this method can provide absolute quantitative measures of blood flow, which are not readily obtained by other modalities. Pseudocontinuous ASL (pCASL), in particular, has been recommended as the methodology of choice for clinical ASL [4], but no such recommendations currently exist for mice. Importantly, many potential therapeutic strategies for brain tumours are initially tested in mouse models. Consequently, there is a need for accurate methods of measuring preclinical brain and tumour blood flow, reflecting those used in the clinic, to enable assessment of likely therapeutic efficacy.

Despite initial reports developing ASL in rodents [5, 6], most implementations have focussed on clinical ASL. Many challenges exist in the adaptation of pCASL MRI for preclinical use, including higher blood flow velocities in the feeding arteries, smaller brain size, susceptibility artefacts from neighbouring air spaces, and B_0_ inhomogeneities in high-field preclinical systems. Implementations of ASL in mice have historically produced CBF values that are semiquantitative [7–9], single slice [10], or vary with the sequence [11–13] and body position [14] used. Moreover, the mean brain CBF values obtained are often unphysiologically high (>200 mL/100 g/min) [11, 15–17] and show little concordance with gold-standard autoradiography measurements (*ca.* 100 mL/100 g/min) in the normal mouse brain [18–22]. These values may be influenced by the use of anaesthetics, which at high concentrations can increase CBF dramatically [23, 24]. Whilst some preclinical studies have validated their ASL measurements using autoradiography [25–27] or microspheres [28], pCASL has not yet been validated in the mouse brain. A previous implementation of pCASL to measure CBF in mice not only showed clear advantages but also showed a loss of inversion efficiency stemming from phase offsets in the label-control acquisitions [12].

The use of multiphase pCASL (MP pCASL) has been shown to reduce these errors in both humans [29] and rats [27], but this sequence has not yet been applied in mice. We have recently implemented a multiphase pCASL method for use in the rat brain, which shows high reliability, reproducibility, and accuracy for CBF measurements [27]. However, given the even smaller size of the mouse brain, implementing this sequence in mice remains challenging. Movement artefacts from respiration affect the signal at the base of the brain in particular, and air spaces surrounding the brain produce distortions. Since most preclinical models of primary brain tumours and brain metastasis are established in mice rather than rats, developing this method further for robust and reliable application in mice is essential.

Thus, the primary aim of this study was to optimise and validate MP pCASL MRI for measuring CBF in naïve mice. Subsequently, we assessed the sensitivity of the optimised MP pCASL sequence to changes in blood flow in early stage tumours, using mouse models of secondary brain cancer (metastasis) and primary glioma.

## 2. Materials and Methods

### 2.1. Animals

Female BALB/c mice (Charles River, UK), 7–10 weeks old, were used in all naïve and metastasis model experiments. Female SCID mice (Charles River, UK), 7–10 weeks old, were used in all primary brain tumour model experiments. All animal experiments were approved by the UK Home Office (Animals (Scientific Procedures) Act 1986) and conducted in accordance with the University of Oxford Policy on the Use of Animals in Scientific Research and the ARRIVE guidelines [30]. Animals were housed in individually ventilated cages under a 12-h light/12-h dark cycle with food and water ad libitum. All animals were housed in cages of 4–6 animals.

### 2.2. Carotid Blood Flow Measurements

Mice (*n*=4) were anaesthetised with 3% isoflurane in O_2_. Temperature was monitored and maintained at 37.0 ± 0.5°C throughout with a feedback heating system. Respiration was monitored and maintained between 30 and 80 breaths per minute. An electrocardiogram was also acquired. Using a Vevo 3100 ultrasound (Visualsonics, Amsterdam, Netherlands) and a mouse cardiology probe (MX550D), a respiration-gated 3D ultrasound image was acquired across the whole neck. The left carotid artery was located, and 10 Power Doppler blood flow clips of 30 s duration were acquired per mouse at respiration rates across the range of 30–80 breaths per minute, achieved by varying the concentration of isoflurane between 3 and 1%. Carotid blood flow values from the Power Doppler trace were extracted using an open-source plot digitiser [31], and values representing the systolic and diastolic flow velocities were used in pCASL simulations to determine labelling efficiency.

### 2.3. Labelling Efficiency Simulations

Bloch simulations were carried out to simulate labelling efficiency over a range of different gradient strengths, radiofrequency (RF) powers, labelling plane thicknesses, and blood flow velocities, as described by Okell [32]. We conducted Bloch simulations whilst varying *G*_max_ and G_mean_ (*G*_mean_ = 0.05 × *G*_max_ in our MP pCASL sequence) gradient strengths, equivalent to converted labelling plane thicknesses of 1–10 mm in 0.5 mm steps. Blood flow velocities of between 1 and 20 cm/s with steps of 1 cm/s were modelled with *T*_1blood_ = 2.1 s, *T*_2blood_ = 0.033 s (based on ex vivo rat blood measurements at 9.4 T) [27], and RF pulse amplitudes of 1–10 *µ*T with steps of 0.5 *µ*T for a train of 600 *μ*s Hanning-shaped pulses, beginning every 1.2 ms. Simulations of the theoretical maximum saturation for different label durations were also carried out, and data are available in the Supplementary Information.

### 2.4. MP pCASL Optimisation

MP pCASL experiments were performed on a separate cohort of mice (*n*=4) using a 9.4 T MRI spectrometer (Agilent Technologies Inc., Santa Clara, USA) with a 26 mm volume transmit-receive birdcage RF coil (Rapid Biomedical GmbH, Rimpar, Germany). Mice were anaesthetised with 3% isoflurane in 30% O_2_: 70% N_2_ and positioned in a custom-built cradle in the volume coil. Mouse temperature was monitored and maintained at 37.0 ± 0.5°C with a rectal probe and feedback heating system. Respiration was monitored using a pressure balloon, and breathing rate was maintained at approximately 60 breaths per minute by adjusting isoflurane concentration; stable breathing was typically maintained with an isoflurane concentration of 1.2–1.5%. The respiration trace was recorded and used to inform respiratory triggering.

Zero- and first-order shimming was conducted manually using a PRESS sequence on a voxel encompassing the brain. The EPI readout was acquired with the following parameters: 2-shot spin-echo encoding, relaxation time (TR) = 4.0 s, echo time (TE) = 18.32 ms, spectral width (SW) = 250 kHz, field of view (FOV) = 20 × 20 mm, matrix size = 64 × 64, slice thickness = 1 mm, and number of slices = 8, with the anterior slice positioned just posterior to the olfactory sulcus. Respiratory triggering (with an associated variable post-trigger delay) was also employed to reduce signal fluctuation at the base of the brain caused by respiratory motion.

**Figure 1:**
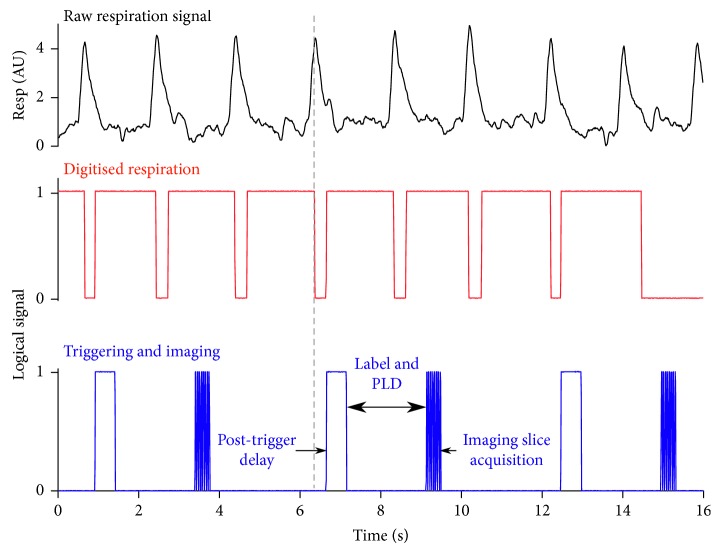
Optimisation of EPI readout using respiratory triggering to acquire the imaging readout during the plateau phase of respiration, reducing signal fluctuation at the base of the brain. Black trace = raw respiration trace; red trace = digitised respiration trace which acts as the trigger signal; and blue trace = readout of the post-trigger delay period, followed by the imaging slice acquisition. A variable encoding the post-trigger delay was implemented in the sequence to allow adjustment for changing respiration rates.

The MP pCASL parameters were optimised for use in mice. The MP pCASL labelling was implemented with a labelling train consisting of a set of slice-selective RF/gradient pulse pairs followed by a slice-refocusing gradient pulse; RF pulse duration 600 *μ*s, Hanning shape with effective flip angle (FA) = 40°, and slice-refocusing gradient pulse duration 600 *μ*s. The phase of these RF pulses was arrayed from 0° to 315° with incremental steps of 45°: 0°, 45°, 90°, 135°, 180°, 225°, 270°, and 315°. The labelling plane was oriented at −10° relative to the axial plane in order to be perpendicular to the carotid and vertebral arteries (Figure 1). The labelling pulse train was followed by a gradient crusher to remove residual transverse magnetisation, a postlabel delay (PLD) to enable labelled blood water spins to travel to the brain, and the EPI readout. Using these parameters and the variable respiration trigger, the duration of the scan varied from 3 minutes 12 seconds to a maximum of approximately 5 minutes. Labelling train duration was varied between 0.4 and 5.0 s (TR increased accordingly from 4–7.6 s) and PLD between 10 and 1000 ms, to optimise the MP pCASL signal.

### 2.5. Validation of MP pCASL by Autoradiography

For comparison of MP pCASL data with gold-standard autoradiography CBF measurements, naïve mice (*n*=4) were anaesthetised using the same setup and isoflurane concentrations as for MRI described above. MP pCASL was performed on each animal on the day before autoradiography, owing to restrictions concerning the use of radioisotopes in certain rooms. Care was taken to ensure breathing rate and isoflurane concentration, and duration of anaesthesia prior to measurements was as similar as possible between the ASL and autoradiography sessions. For the MP pCASL MRI, a labelling pulse duration of 0.9 s and a postlabel delay of 0.4 s seconds was used with the parameters described above.

Autoradiography was performed using the method described by Maeda et al. [19]. Mice were injected intraperitoneally with 0.15 *µ*Ci of 4-iodo-*N*-methyl-[^14^C]antipyrine (Hartmann Analytic, Germany, specific activity 55 mCi/mmol), and 2 minutes later, they were injected intraperitoneally with an overdose of pentobarbitone then immersed in isopentane on dry ice until frozen. The frozen brain was extracted, and 20 *µ*m sections were collected at 200 *µ*m intervals and dried at 60°C for 10 min. These sections were exposed to film for 24 hours alongside calibrated radioactive standards, and films were scanned (Carestream Kodak BioMax MR Film; standards: 0–35 *µ*Ci/g, ARC; Expression 10000XL transmittance scanner, Epson, UK). Scanned films were background subtracted and calibrated against the standards. Absolute CBF was then calculated using the equation as described by Sakurada et al. [33]. Blood sampled from the frozen heart at end-point was used to calculate the final arterial tracer concentration using autoradiography. For calculation of regional CBF values, the autoradiography images were perspective transformed, aligned to the MR images, and down-sampled to the same resolution as the MP pCASL images (64 × 64 pixels). Subsequently, regional masks were created.

### 2.6. MP pCASL Data Analysis

ASL data analysis and perfusion quantification were performed using a custom version of the BASIL toolbox from the FMRIB Software Library (http://www.fmrib.ox.ac.uk/fsl/BASIL). The raw multiphase data were initially fitted to a modified Fermi function [27] (*α*=70, *β*=19) to produce a raw phase map. The phase values were then smoothed and clustered using supervoxel clustering [34] to produce regions of interest (ROIs) for each supervoxel phase cluster. The raw multiphase data were combined with the supervoxel ROIs to produce high SNR means of the optimal phase for each supervoxel. The high SNR supervoxel data were then fitted to the Fermi function again to produce a high-precision phase map, which could be used to calculate the final CBF map.

Perfusion quantitation was performed according to the kinetic model of Buxton et al. [35] and calibration with Oxford_asl [36]. Reference scans acquired without labelling were used for absolute quantitation of CBF. Detailed postprocessing methods, including supervoxel clustering, are described by Larkin et al. [27].

### 2.7. Mouse Models of Brain Metastasis and Primary Brain Tumours

Sensitivity of the MP pCASL sequence to changes in tumour blood flow over time was first assessed in a brain metastasis model. Female BALB/c mice were anaesthetised with 3% isoflurane in 30% O_2:_ 70% N_2_O, and focally microinjected using a finely drawn glass microcapillary (*ca.* 75 *µ*m tip diameter) with 5000 4T1-GFP, metastatic murine mammary carcinoma cells in 0.5 *µ*L sterile saline into the left striatum (+0.5 mm anterior, +2 mm lateral, 2.5 mm depth relative to bregma). Mice underwent MRI at day 7 (*n*=3), 14 (*n*=5), 21 (*n*=6), 28 (*n*=8), or 35 (*n*=6) after tumour induction; mice were randomly assigned to each time point group.

To further assess the sensitivity of the MP pCASL method, a primary brain tumour model was also used. Female SCID mice were anaesthetised and injected in the left striatum, as above, with 5000 U87 human glioma cells. Mice underwent MRI at day 14 (*n*=10) or 28 (*n*=6), after tumour induction.

In addition to MP pCASL acquisitions, *T*_1_ and *T*_2_ maps, angiography, and *T*_1_ and *T*_2_ weighted anatomical scans were acquired for each mouse. Time-of-flight angiography was acquired with TR = 30 ms, TE = 1.78 ms, FA = 30°, matrix size = 128 × 128 × 128, and FOV = 20 × 20 × 30 mm. A *T*_1_-weighted spin-echo multislice anatomical scan was acquired with TR = 0.55 s, TE = 20 ms, number of averages = 2, and matrix size = 256 × 256. A *T*_2_-weighted fast spin-echo multislice anatomical scan was acquired with TR = 3.5 s, echo spacing = 15 ms, effective TE = 60 ms, echo train length = 4, and matrix size = 256 × 256; using the same slice plan and FOV as the MP pCASL. A contrast-enhanced scan was obtained 5 minutes following intravenous injection of 30 *µ*L (15 *µ*mol, approximately 0.75 mmol/kg based on a 20 g mouse) Gadodiamide (Omniscan, GE Healthcare) using the *T*_1_-weighted scan parameters described above.

Following imaging, mice underwent transcardial perfusion-fixation with 0.9% heparinised saline followed by 20 mL periodate-lysine-paraformaldehyde solution containing 0.025% glutaraldehyde, and the brains were collected for histology.

### 2.8. Immunohistochemistry

Sections were stained for the blood vessel marker CD31 (AF3628, R&D systems, Abingdon, UK) according to the method described by Andreou et al. [37]. Microvessel density (vessel area fraction) was quantified for the core and rim regions of the entire tumour, and also for the contralateral striatum, as the percentage of area covered using the “Positive Pixel Count 2004-08-11” algorithm in Imagescope (Leica Biosystems). The parameters used for perfusion-fixed tumour model 10µm sections were moderately stained pixel intensity between 202 and 185 and strongly stained pixel intensity lower than 10. The parameters used for postfixed 20 *µ*m autoradiography sections were moderately stained pixel intensity between 180 and 171, and strongly stained pixel intensity lower than 53.

### 2.9. Statistics

All data are reported as group mean ± group standard deviation. Differences between groups were determined using two-tailed paired *t*-tests or one-way ANOVA (regional and histology analysis), or two-way repeated measures (paired) ANOVA (time-course analysis) followed post hoc by the Bonferroni multiple test correction. An f-test was used to assess heterogeneity between groups.

## 3. Results

### 3.1. MP pCASL Sequence Optimisation

Blood velocity in the carotid artery was measured to inform Bloch simulations of pCASL. The mean blood velocity in the carotid artery was found to be 124 ± 17 mm/sec, and the maximum blood velocity was found to be 275 ± 58 mm/sec. Mean blood velocity was consistent between animals, but varied with the respiration rate in 3 of 4 animals (linear regression, slope = 0.3–1.4). Maximum blood velocity varied between animals (range = 214–556 mm/sec), and also varied with the respiration rate in 3 of 4 animals (linear regression, slope = 2.0–7.6). Using these values to inform the range of blood velocities in the Bloch simulations, a 2 mm label thickness was chosen as it provided sufficient theoretical inversion (labelling) efficiency (78.5%) at the RF pulse amplitude used in our system (5 *µ*T) and average blood velocity of 124 mm/s, and lies within the linear portion of the feeding arteries in the neck.

Respiratory triggering, whereby the EPI readout was acquired during the plateau phase of respiration, was found to reduce signal fluctuation at the base of the brain (Supplementary Figure 1). The respiratory triggering also significantly increased the signal-to-noise ratio (SNR) of the MP pCASL data (15.1 ± 5.9) compared wih the untriggered sequence (6.5 ± 1.5, *p* < 0.05; Supplementary Figure 2). Finally, a variable post-trigger delay was encoded in the sequence to allow adjustment for changing respiration rates in subsequent scans (Figure 1).

**Figure 2:**
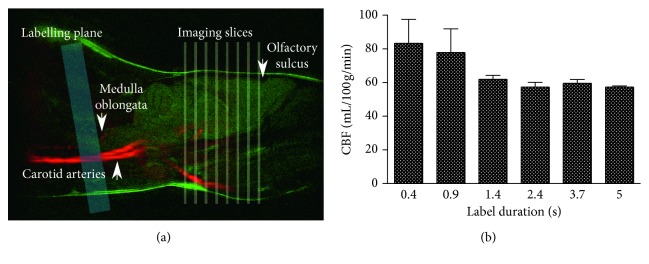
Optimisation of MP pCASL bolus duration. (a) A−10° labelling plane (blue) relative to the axial imaging plane (yellow) was optimal, as this was perpendicular to the carotid and vertebral arteries visible by angiography (red). Label plane placement posterior to the medulla oblongata, visible on a sagittal *T*_2_ weighted anatomical image (green), enabled consistent label plane location between animals, while not interfering with imaging slices (yellow). (b) No significant differences were found in calculated CBF across the range of label duration times tested (*n*=3).

Following angiography, a labelling plane positioned at −10° relative to the axial plane was found to be perpendicular to the carotid and vertebral arteries. Label placement just posterior to the medulla oblongata enabled consistent labelling plane placement between animals using a distinct anatomical landmark, and is posterior to the vertebral flexure of the carotid arteries (Figure 2(a)).

All labelling pulse train durations tested yielded CBF values close to the physiological range expected from the literature (Figure 2(b)), and no significant differences in calculated CBF values were found between the different durations. Similarly, phase maps were stable across labelling durations. Thus, as a compromise between ensuring sufficient labelling (SNR simulations described in Supplementary information) and keeping scan time as short as possible, a label duration of 0.9 s was chosen.

**Figure 3:**
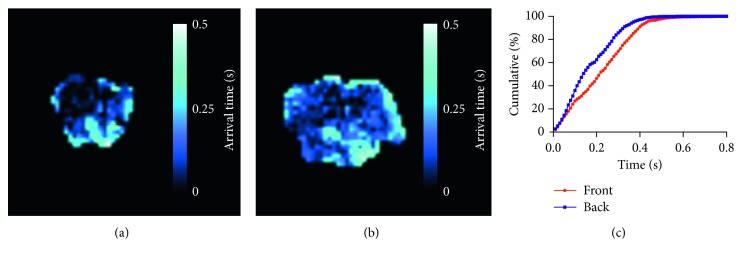
Optimisation of postlabel delay (a–b). Multi-PLD scans were acquired to measure arterial transit time at the front and back of the brain. Example maps of bolus arrival time in (a) anterior and (b) posterior slices. (c) Arrival maps showed bolus arrival in over 95% of voxels in the anterior slice and 99% of voxels in the posterior slice within 0.4 s (*n*=4).

To allow measurement of bolus arrival time in brain voxels, 12 PLDs from 10 to 1000 ms were acquired, each with 8 phases of labelling RF pulses. Arrival maps showed that the bolus arrived at over 95% of voxels in the most anterior slice and at 99% of voxels in the most posterior slice within 0.4 s (Figure 3(c)). Thus, 0.4 s was chosen as the optimal PLD to reduce signal loss from relaxation.

#### 3.1.1. MP pCASL Validation

**Figure 4:**
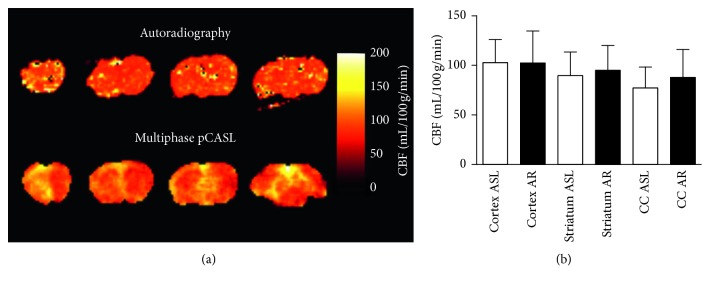
Cerebral blood flow maps and regional values. (a) Representative CBF maps produced using gold-standard autoradiography (top row), and the optimised MP pCASL sequence (bottom row). (b) Regional CBF values as measured by MP pCASL and autoradiography (AR). Average CBF values were 96 ± 18 mL/100 g/min across the whole brain using MP pCASL, and 101 ± 32 mL/100 g/min using autoradiography. No significant differences were found between MP pCASL and autoradiography CBF measurements either for whole brain, or by region. CC–corpus callosum.

Use of the optimised MP pCASL sequence in naïve mice yielded measured CBF values of 96 ± 18 mL/100 g/min on average across the brain (Figure 4(a)), 103 ± 23 mL/100 g/min in the cortex, 90 ± 24 mL/100 g/min in the striatum, and 77 ± 21 mL/100 g/min in the corpus callosum. Using the gold-standard autoradiography method, we measured CBF to be 101 ± 32 mL/100 g/min across the whole brain, a difference of <6% when compared with MP pCASL results across the same region (Figure 4(a)). Average CBF values using autoradiography were 102 ± 32 mL/100 g/min in the cortex, 95 ± 25 mL/100 g/min in the striatum, and 87 ± 28 mL/100 g/min in the corpus callosum. No significant differences were found between MP pCASL and autoradiography CBF measurements (Figure 4(b)). Blood vessel density was calculated from histological sections stained for CD31 in these animals, yielding values of 3.0 ± 0.6%, 2.2 ± 0.8%, and 0.8 ± 0.3% for the cortex, striatum, and corpus callosum, respectively (Supplementary Figure 3). Although both CBF and vessel density appeared to decrease across the three regions studied (cortex > striatum > corpus callosum), no significant correlation between vessel density and CBF was found.

#### 3.1.2. Mouse Model of Brain Metastasis

Application of the MP pCASL sequence in mice with intracerebral metastases showed a significant, 16%, decrease in CBF in the tumour-bearing striatum (84 ± 16 mL/100 g/min) compared with the contralateral (normal) striatum (99 ± 24 mL/100 g/min) at day 35 (ANOVA *p* < 0.05; post hoc Bonferroni test *p* < 0.05). The mean absolute reduction in CBF in the tumour-bearing striatum was 15.3 mL/100 g/min. No differences were found between hemispheres at other time points. All tumours were confirmed histologically, and the mean tumour diameter measured by histology at day 35 was 2.14 ± 0.53 mm.

**Figure 5:**
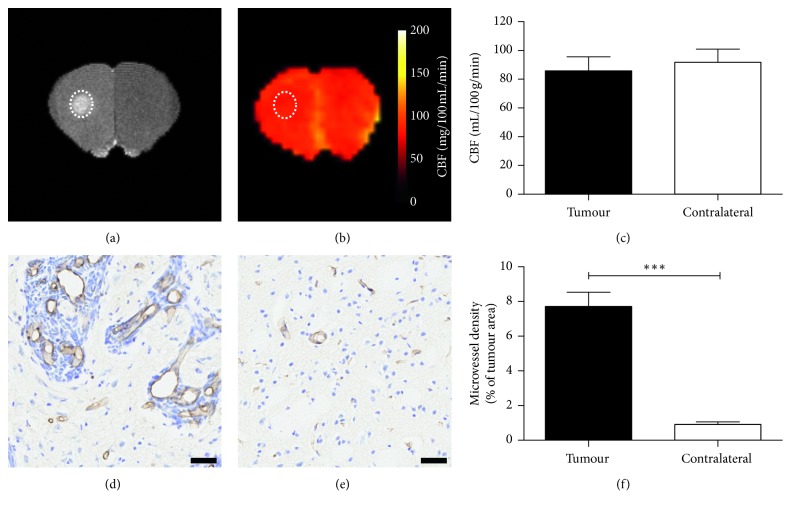
Application of the MP pCASL sequence in mice with intracerebral 4T1-GFP metastatic tumours showing gadolinium enhancement. (a) *T*_1_-weighted image showing enhancement (dotted outline) of metastatic foci. (b) CBF maps with the same ROI indicated. (c) Graph showing CBF values in tumour and contralateral regions; no significant decrease in perfusion is evident (d-e). Histological comparison of microvessel area fraction in tumour (d), and contralateral (e) regions. Scale bars = 20 *µ*m. (f) Graph showing quantitation of microvessel area fraction in tumour and contralateral striatum regions; a significant increase in microvessel area fraction is evident in tumour compared with normal tissue in the contralateral striatum (*n*=7). ^*∗∗∗*^ *p* < 0.01.

In a subset of animals from days 21–35 (*n*=7), the striatum exhibited gadolinium enhancement throughout the area of metastatic foci and, in these animals, no difference in CBF was evident between the tumour (86 ± 26 mL/100 g/min) and contralateral striatum (92 ± 24 mL/100 g/min; Figures 5(a)–5(c). Analysis of microvessel density within the metastatic foci showed an increased vessel area fraction (7.7 ± 2.2%; Figure 5(d)) in the enhancing tumour compared with normal tissue in the contralateral striatum (0.9 ± 0.4%, *p* < 0.0005; Figures 5(e)–5(f)).

**Figure 6:**
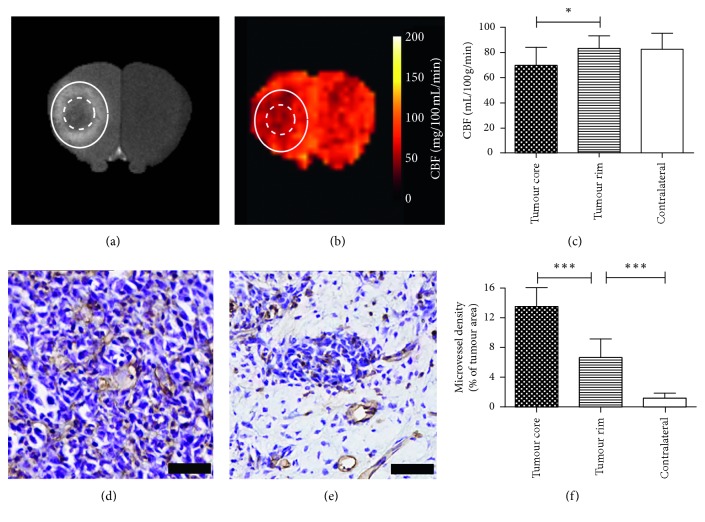
Application of the MP pCASL sequence in mice with intracerebral 4T1-GFP metastatic tumours showing a gadolinium enhancing rim and a nonenhancing core region. (a) *T*_1_-weighted image showing core (nongadolinium-enhancing central regions; dotted outline) and rim (gadolinium-enhancing regions; solid outline) of metastatic foci. (b) CBF maps with the same ROIs indicated. (c) Graph showing CBF values in core, rim, and contralateral regions; a significant decrease (*n*=6) in perfusion is evident between the core and rim regions of tumours (d-e). Histological comparison of microvessel area fraction in core (d) and rim (e) regions. Scale bars = 20 *µ*m. (f) Graph showing quantitation of microvessel area fraction in core, rim, and contralateral striatum regions; a significant increase in vessel area fraction is evident in core regions compared with both the rim regions (*n*=6) and normal tissue in the contralateral striatum. ^*∗*^ *p* < 0.05; ^*∗∗∗*^ *p* < 0.0001.

However, in a further, distinct subset of animals from days 28 and 35 (*n*=6), a gadolinium-enhancing tumour rim was evident surrounding a nonenhancing tumour core. Analysis of these animals showed a significant reduction in blood flow in the core of the tumour region (69 ± 14 mL/100 g/min) compared with the rim (83 ± 10 mL/100 g/min, *p* < 0.05; Figures 6(a)–6(c). No difference was found between the enhancing rim of the tumour and normal tissue in the contralateral striatum (82 ± 13 mL/100 g/min; Figure 6(c)). The mean absolute reduction in CBF in the tumour core compared with that in the tumour rim was 13.4 mL/100 g/min. The tumour voxels (including core and rim) showed significantly greater heterogeneity, as demonstrated by higher intra-ROI voxel variance, than the contralateral striatum (F-test, *p* < 0.001). Analysis of microvessel density within the metastatic foci showed a greatly increased vessel area fraction (13.5 ± 2.5%) in the nonenhancing tumour core compared with the tumour rim (6.6 ± 2.5%, *p* < 0.0001; Figures 6(d)–6(f)), which in turn showed greater vessel area fraction than the normal tissue in the contralateral striatum (1.2 ± 0.7%, *p* < 0.0001; Figure 6(f)). Correlation analysis between microvessel density and CBF measured by MP pCASL MRI revealed a trend towards negative correlation in both tumour core and rim, but this did not reach significance (Supplementary Figure 4).

#### 3.1.3. Mouse Model of Glioma

**Figure 7:**
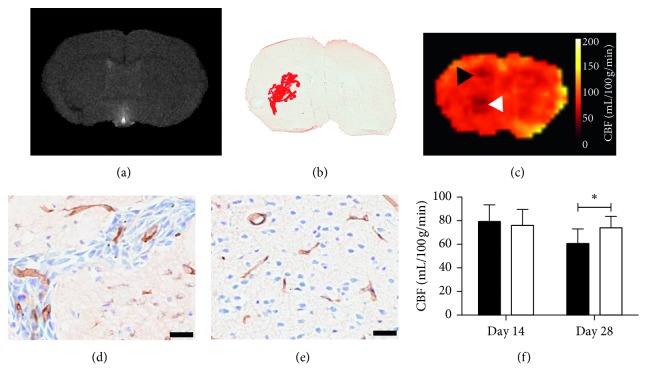
Application of the MP pCASL sequence in mice with intracerebral U87 glioma at the day 28 time point. (a) *T*_1_-weighted image showing no evidence of gadolinium enhancement in the tumour injected (left) hemisphere. (b) Immunohistochemical image showing an overlay of tumour area (red) combined from tissue sections spanning 500 *µ*m corresponding to the MRI slice in (a). (c) CBF maps showing reduction in local CBF (arrowheads) (d-e). Immunohistochemical image showing CD31 staining of vessels (brown) in tumour-injected striatum (d) and contralateral striatum (e). (f) Graph showing CBF values in the tumour-bearing (black) and contralateral (white) striatum. CBF is reduced in the tumour-bearing compared with the contralateral striatum at day 28 time point (*p*=0.02). Scale bars = 20 *µ*m.

Application of the MP pCASL sequence in mice injected intracerebrally with U87 glioma cells showed a significant, 19%, reduction in CBF in the tumour-bearing striatum (61 ± 12 mL/100 g/min) compared with the contralateral striatum at day 28 (74 ± 10 mL/100 g/min, *p* < 0.05; Figures 7(a)–7(c)). The mean absolute reduction in CBF in the tumour-bearing striatum was 13.3 mL/100 g/min. Only two tumours showed gadolinium enhancement, and no tumours showed nonenhancing core regions. As for the metastasis model, analysis of microvessel density within the day 28 tumours showed an increased vessel area fraction (10.7 ± 2.9%; Figure 7(d)) in the tumour compared with the normal tissue in the contralateral striatum (3.6 ± 0.5%, *p* < 0.05; Figures 7(e)–7(f)). All tumours were confirmed histologically, and the mean tumour diameter measured by histology at day 28 was 1.20 ± 0.52 mm.

## 4. Discussion

Pseudocontinuous ASL is the recommended MRI sequence for clinical measurements of cerebral blood flow, and multiphase ASL sequences have many advantages in compensating for off-resonance effects that alter labelling efficiency, thus improving the quality and reliability of CBF maps produced. Despite these advantages, no studies have been published implementing multiphase pCASL in mice. We have now demonstrated that our optimised MP pCASL sequence provides reliable and accurate measures of CBF in naïve mice in vivo and is sensitive to changes in tumour blood flow.

In this study, our aim was to produce MP pCASL measurements that can be used to obtain CBF maps in mice that replicate blood flow values obtained using the gold-standard approach of autoradiography. Our results showed good concordance with autoradiography and were in accord with previous autoradiography findings [19], including those comparing ASL with autoradiography [22, 27]. As anticipated, the use of multiphase pCASL reduced the errors seen in the previous implementation of label-control pCASL in mice, owing to loss of inversion efficiency [12]. As an alternative to the MP pCASL approach described here, phase correction can also be applied using prescans [38]. However, this method requires processing during scan time rather than post hoc. For this reason, the MP pCASL approach offers an easier implementation and potentially more time efficient approach for the measurement of CBF.

The inclusion of a respiration trigger in the sequence reduced signal fluctuation at the base of the brain and improved image quality. However, this technique does result in a variable TR between phase acquisitions. As the TR becomes longer than the minimum with triggering, it is expected that there will be no effect on the next acquisition. Extended TR values of up to 20 s have been tested with this sequence and have shown no effect on data quality (data not shown). The use of respiratory triggering also relies on a reasonably stable respiratory cycle, which was reliably achieved at ∼60 breaths per minute with 1.2–1.5% isoflurane concentrations for up to two hours.

Our data show less regional variation in CBF than other autoradiography studies in mice, which may, at least in part, reflect differences in mouse strain and anaesthetic regime used [19, 21, 39]. Gaseous anaesthesia, in particular, is known to have region specific effects on CBF, and in rats the caudate/putamen shows a much greater (∼70%) increase in CBF than in the cortex (∼20%) under isoflurane-anaesthetised conditions compared with awake animals [23]. Consequently, regional differences in CBF under isofluorane anaesthesia are greatly reduced [23], and it is likely that this underlies the reduced regional variation in CBF observed in the current study. The reduced regional variation was observed in both MP pCASL and autoradiography data, supporting the above conclusion. To reduce the effects of isoflurane on our CBF measurements, we used the lowest possible concentration of isoflurane in order to achieve a stable breathing rate of ∼60 breaths per minute.

The MP pCASL technique was found to be sensitive to changes in tumour perfusion in mouse models of both brain metastasis and glioma at later time points. The technique was also sensitive to perfusion heterogeneity within metastatic tumours, showing significantly reduced (but not absent) perfusion in the nonenhancing tumour core compared with normal tissue in the contralateral striatum and increased heterogeneity in perfusion compared with the contralateral striatum. As autoradiography has only been used to validate CBF measurements in the naïve brain, it is possible that differences in tumour arterial transit time or tissue relaxation time may affect the CBF quantification from MP pCASL data in these tumour-bearing animals. However, these results are in line with other preclinical primary brain tumour perfusion studies, which showed reduced perfusion in the tumour core [7, 40–42]. The reduction in tumour blood flow observed in the current study was considerably smaller than other (primary) tumour perfusion studies. The majority of previous studies have investigated large tumours (>5 mm diameter in mice) [7, 9, 40, 42], which often have a large necrotic core with few blood vessels [7, 43] resulting in a substantial perfusion deficit. In contrast, in both the metastasis and glioma models used here, the tumour foci were considerably smaller (1–2 mm diameter) and more diffusely growing. Moreover, histological analysis demonstrated that the core regions, rather than being necrotic and devoid of vessels, in fact contained large numbers of blood vessels. Such tumours would be ideal candidates, clinically, for treatment with vascular modifying agents, and the ability to quantitatively measure changes in blood flow in such small tumours could be of considerable use in preclinical testing.

Tumours have a number of distinct vascular phenotypes, each of which may confer changes in blood flow. One such phenotype, which is present in our model, is that of a highly branched network of nonfunctional angiogenic vessels. In this case, despite an increase in the density of vessels, a decrease in CBF may be observed in this region, owing their highly branched and dysfunctional nature. Another vascular phenotype often seen in solid tumours is the rapid growth of the tumour faster than the vasculature can keep up, resulting in a tumour core with low vascularity, and hence a decreased CBF. Both of these phenotypes are characterised by a decrease in CBF, despite the differences in tumour vascularity.

Gadolinium contrast enhancement on MRI reports on both vessel permeability and blood flow, and a lack of gadolinium enhancement in tumour core regions is typically taken to reflect tissue necrosis and complete absence of vessels. In contrast, blood vessel density measurements provide no information on whether vessels are perfused, but only whether they are present. However, in both models used in the current study, although core tumour areas showed significant reductions in blood flow in areas devoid of gadolinium enhancement, histological assessment revealed a significant increase in microvessel density and only very small areas of necrosis. Together, these findings suggest that these core vessels are either structurally abnormal or have abnormal flow patterns, as is common in angiogenic tumour vessels [44–46], and that MP pCASL measurements may complement histology and contrast-enhanced MRI to provide useful insights into vessel patency and tumour blood flow.

Interestingly, the rim of the metastatic tumour area also showed an increase in microvessel density histologically, but in this region, CBF measured by MP pCASL MRI was normal. Similarly, in those tumours showing enhancement throughout the tumour core, microvessel density was increased, but no change in CBF was evident. These findings suggest that MP pCASL MRI identifies heterogeneity in tumour blood flow that could not be predicted from histological assessment of vascularity alone. Thus, by identifying areas of reduced (but not absent) flow, MP pCASL MRI may enable identification of brain tumours in which vascular normalising therapy or antiangiogenic therapy could be beneficial prior to, or in conjunction with, radiotherapy.

## 5. Conclusions

Multiphase pCASL MRI has been successfully implemented and validated for imaging cerebral blood flow in naïve mice and has been shown to overcome the limitations inherent in standard nonmultiphase ASL methods. The data show high reproducibility in CBF measurements between animals and concordance with gold-standard autoradiography measurements. Further, this imaging approach is sensitive to changes in perfusion in mouse models of both primary and secondary brain tumours, even in very small tumours, and provides insight into tissue blood flow dynamics that complements vessel density measurements and contrast-enhanced MRI. This method could be of high value for noninvasive measurement of brain tumour blood flow prior to and following therapy, increasing the potential for clinical translation.

## Data Availability

The data used to support the findings of this study are available from the corresponding author upon request. The supervoxel clustering and perfusion quantification methods are available at http://www.quantiphyse.org.

## Supplementary Materials

Supplementary Materials Supplementary Figure 1 (video): example MP pCASL images with (A) and without (B) respiratory triggering. Respiratory triggering reduces signal fluctuation at the base of the brain. Supplementary Figure 2: signal-to-noise ratio (SNR) of the MP pCASL sequence with and without respiratory triggering (*n*=4, ^*∗*^ *p* < 0.05). Supplementary Figure 3: microvessel density of brain regions in naïve animals used for autoradiography (*n*=4). ^*∗*^ *p* < 0.05; ^*∗∗*^ *p* < 0.01 Supplementary Figure 4: plot showing correlations between CBF and microvessel density in a subset of animals (*n*=6) with a gadolinium enhancing rim and nonenhancing core structure. Linear regression lines for each dataset are plotted, showing a trend towards an inverse correlation between CBF and microvessel density for core and rim regions. Supplementary Figure 5: change in theoretical signal saturation over a range of label durations.
