# Supplementary material for: Sensitivity of Multiphase Pseudocontinuous Arterial Spin Labelling (MP pCASL) Magnetic Resonance Imaging for Measuring Brain and Tumour Blood Flow in Mice
Source: Contrast Media Mol Imaging. 2018 Nov 7;2018:4580919. doi: 10.1155/2018/4580919 (PMC6247770; doi:10.1155/2018/4580919)
Supplement: Supplementary Materials — Supplementary Figure 1 (video): example MP pCASL images with (A) and without (B) respiratory triggering. Respiratory triggering reduces signal fluctuation at the base of the brain. Supplementary Figure 2: signal-to-noise ratio (SNR) of the MP pCASL sequence with and without respiratory triggering (n=4, ∗ p < 0.05). Supplementary Figure 3: microvessel density of brain regions in naïve animals used for autoradiography (n=4). ∗ p < 0.05; ∗∗ p < 0.01 Supplementary Figure 4: plot showing correlations between CBF and microvessel density in a subset of animals (n=6) with a gadolinium enhancing rim and nonenhancing core structure. Linear regression lines for each dataset are plotted, showing a trend towards an inverse correlation between CBF and microvessel density for core and rim regions. Supplementary Figure 5: change in theoretical signal saturation over a range of label durations. [file 4580919.f1.zip › 4580919.f1/Supplementary Material FINAL_CMMI_2502591.docx]

**Supplementary Material**

Supplementary Figure 1 (video): Example MP pCASL images with (A) and without (B) respiratory triggering. Respiratory triggering reduces signal fluctuation at the base of the brain.


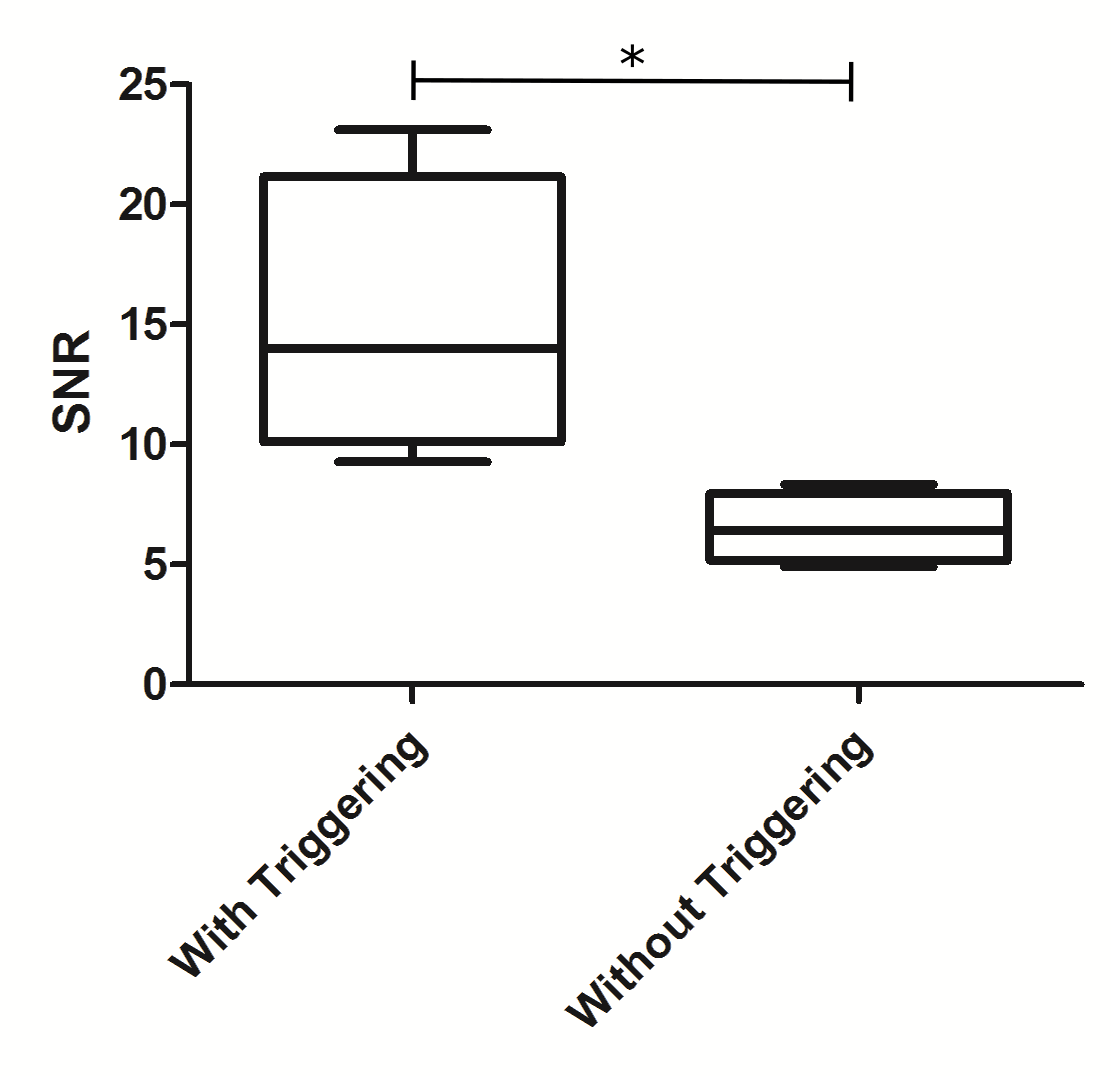


Supplementary Figure 2: Signal-to-noise ratio (SNR) of the MP pCASL sequence with and without respiratory triggering (n = 4, *p < 0.05).


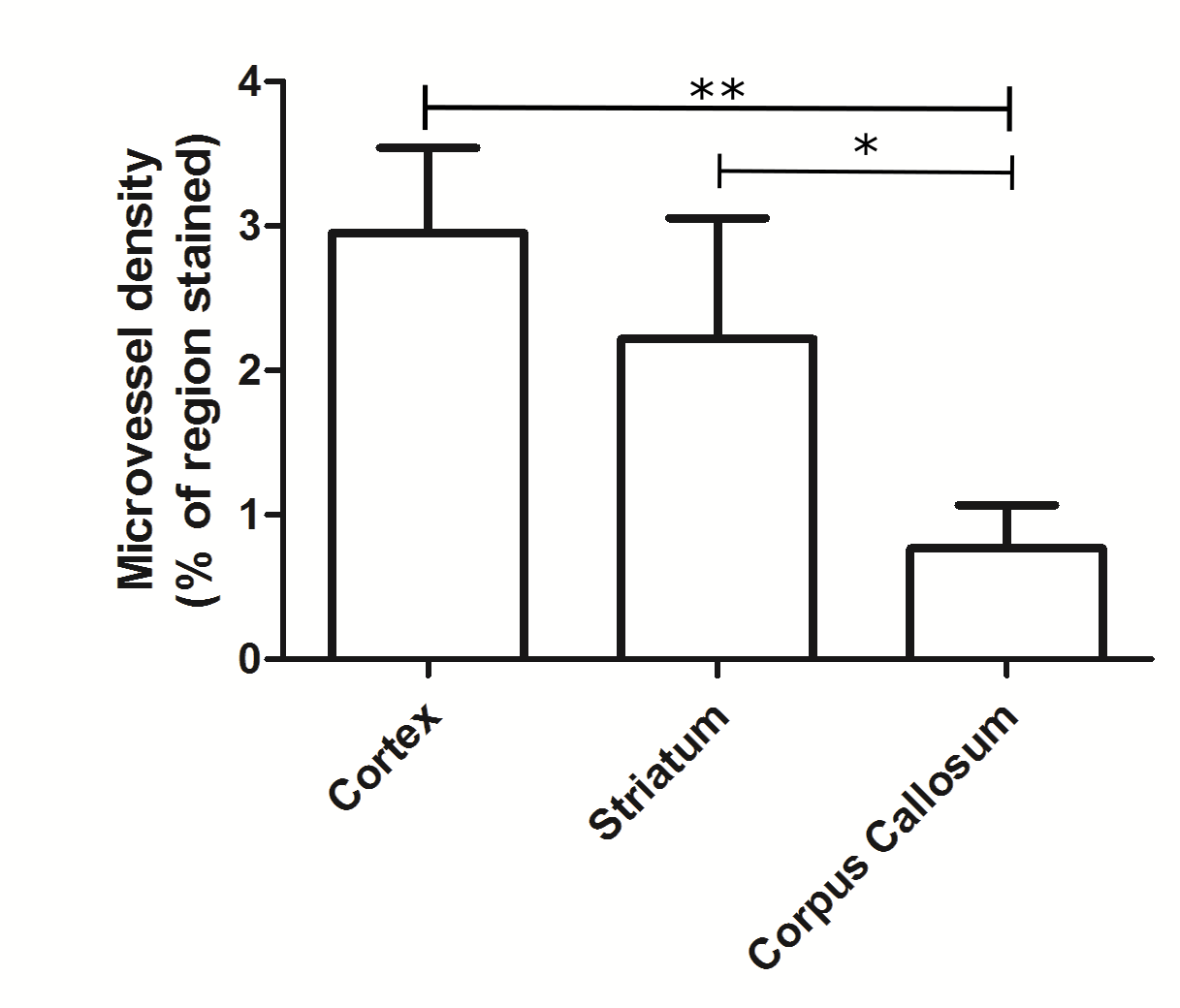


Supplementary Figure 3: Microvessel density of brain regions in naïve animals used for autoradiography (n = 4). * p < 0.05; ** p < 0.01


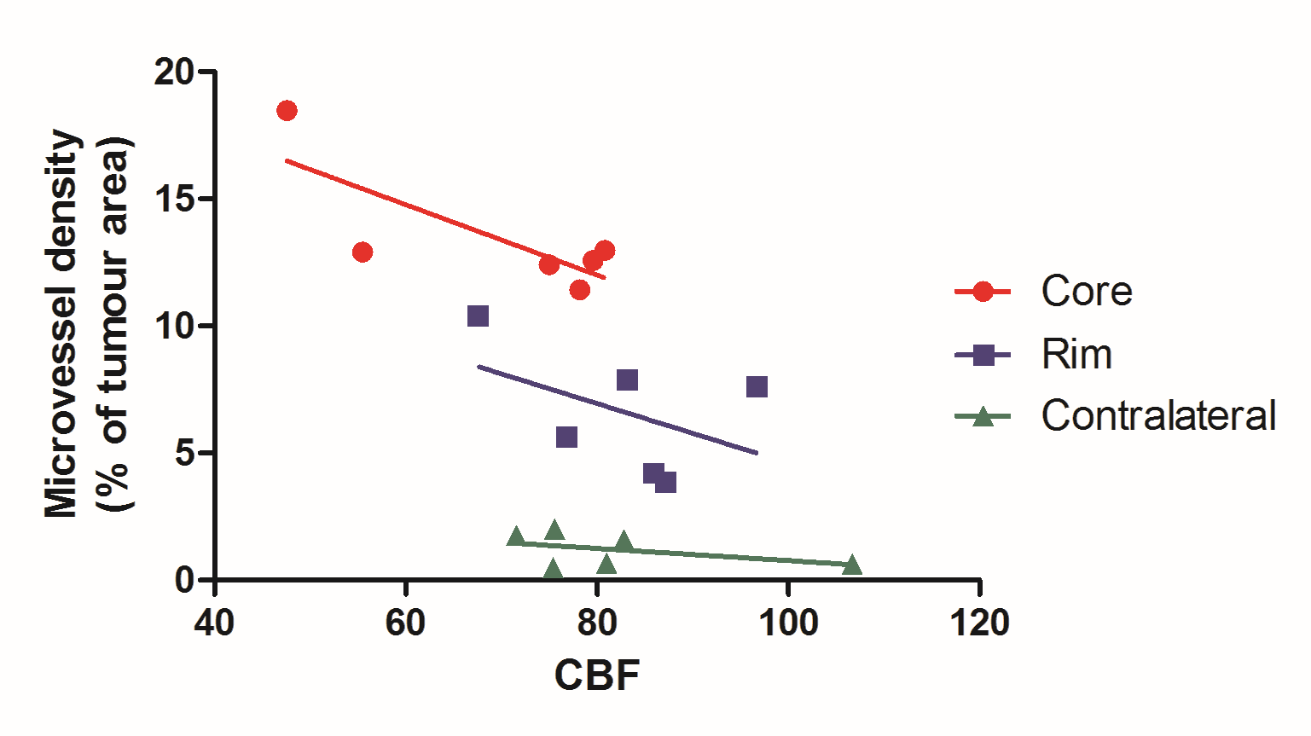


Supplementary Figure 4: Plot showing correlations between CBF and microvessel density in a subset of animals (n = 6) with a gadolinium enhancing rim and non-enhancing core structure. Linear regression lines for each dataset are plotted, showing a trend towards an inverse correlation between CBF and microvessel density for core and rim regions.

Supplementary Methods and Results:

Simulations to estimate the effect of label duration on the net magnetisation signal were carried out. We modelled saturation recovery of labelled water in the brain by assuming full saturation of the in-flowing blood from the labelling plane, an instant transfer of labelled water to the imaging region and relaxation according to the blood T_1_. Under such conditions, tissue saturation increases as a function of label duration, eventually reaching a plateau whose level is dictated by the T_1_ recovery equation:

$$\text{M = }M_{0}\text{ }\left( 1- e^{{-t}/{T1}} \right)\text{ }$$

Using these theoretical maximum signal change values, and the SNR data from respiration triggered MP pCASL, SNR values with 95% confidence intervals were calculated (Supplementary Figure 5) for label durations of 0.1 – 5 seconds.

**
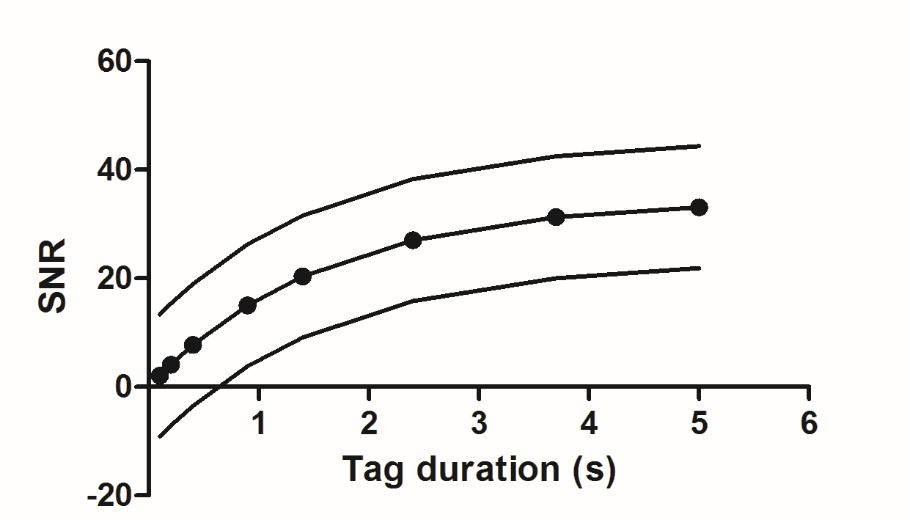
**

Supplementary Figure 5: Change in theoretical signal saturation over a range of label durations.

A tag duration of 0.9 s was the lowest duration tested with a 95% confidence interval of an SNR above zero. As no significant differences in CBF were found with different label durations, a label duration 0.9 s was chosen in order to minimise scan time.
